# Experimental Analysis of the Function of a Window with a Phase Change Heat Accumulator

**DOI:** 10.3390/ma13163647

**Published:** 2020-08-17

**Authors:** Lech Lichołai, Michał Musiał

**Affiliations:** The Faculty of Civil and Environmental Engineering and Architecture, Rzeszow University of Technology, 35-084 Rzeszów, Poland; mmusial@prz.edu.pl

**Keywords:** innovative phase change material, passive solar system, mathematical model of blurred phase change, heat-storing windows

## Abstract

The article presents the results of long-term field tests and their mathematical analysis regarding the impacts of innovative phase change materials on the energy efficiency of composite windows with various glazing parameters. Research was conducted on six glazing combinations throughout the heating season in a temperate climate in Rzeszów (Poland). The empirical results obtained during the spring months showed an improvement in the monthly heat balance for windows with phase change materials compared to the reference window by as much as 34.09%. In addition, the empirical results allowed the development and verification of a mathematical model describing the transport and distribution of heat within a window with a phase change heat accumulator. The model was made using equations of non-stationary heat flow and an explicit finite difference method using calorimetric thermograms describing the phase change eutectic mixture used in the research. Carrying out the Snedecor–Fischer test proved the statistical adequacy of the developed model in 4 out of 6 tested combinations of glazing units. Good matching of the empirical and theoretical quantities was also confirmed using the quasi-Newton method. The article is a solution to the problem of the effective use of solar energy within transparent building partitions, while presenting a useful mathematical tool that determines potential thermal gains in various climatic conditions.

## 1. Introduction

From the beginning of the creation of buildings, people have sought to illuminate their interiors with sunlight while protecting themselves against changing climatic conditions. This has resulted in the development of the solar architecture trend cited in [1,2], as well as the creation of constantly improving transparent building partitions [2]. Along with the next stages of scientific, technological, and economic development, more attention is being paid to aspects of energy-saving construction, renewable energy sources, and innovative building materials. In modern times, there has been an acyclical increase of ecological awareness in the world, which according to [2] is a consequence of sudden increases in the functioning costs or construction of buildings. In addition, the sudden increase in the human population in the 20th century and the limited materials and energy resources in the world have led to a change in the perception of the relationship between human activity and the natural environment, which allowed the formulation of the Brundtland report (UN 1987), which stated that “meeting the needs of the present cannot compromise the ability of future generations to meet their own needs” [3]. The change in the current attitude to the above-mentioned issues was justified by the fact that, as shown in [3,4], all of the processes for the production of building materials, construction of buildings, and their operation and demolition consumes 40% of the electricity produced, 50% of the total processed mass, and generates 35% of greenhouse gases. This resulted in the life cycle costing (LCC) of buildings being considered during the design process and the issue of building permits for the building or extension of buildings with a correspondingly low value for the building’s primary energy demand [5]. Considering the above economic aspects, the location and availability of renewable energy sources, as well as the nature of the external climate and the dissemination of technology, solar energy is one of the most commonly used renewable energy sources in construction.

According to [6,7,8,9], the use of passive solutions and direct gain systems, such as the innovative modification of transparent building partitions, is one of the possibilities to increase the share of heat gains from solar energy conversion.

Due to their structure and function, transparent building partitions are characterized by high instantaneous efficiency, while being susceptible to changes in atmospheric conditions. This is due to their low heat capacity. On the other hand, by using glazing of various values, depending on the orientation towards the world and the nature of the climate, transmittance, absorption, reflectivity, thermal resistance, and light transmittance, it is possible to partly influence the heat gains and losses generated by a building window [7,10]. Considering the above advantages and disadvantages of transparent construction partitions, one of the solutions affecting the increase of their energy efficiency and improvement, according to [8,9], is the increase of their heat capacity, for example through the use of phase change materials.

The thermal capacity of transparent partitions is increased by isothermal storage of heat absorbed by a phase change material (PCM) at the temperature at which its phase transformation occurs [7,10]. The two basic quantities characterizing the justification for using a given phase change material in a selected place are its phase transition enthalpy ΔH and the temperature range in which it occurs. In the next step, other physical and chemical properties are considered, such as the thermal conductivity, thermal expansion, toxicity, chemical affinity, chemical aggressiveness, polymorphism ability, combustion temperature, and aging ability after many cycles of phase transformations [11]. Phase change materials according to [12,13,14,15,16,17,18] are divided into organic, inorganic, and eutectic mixtures according to their physical and chemical properties.

Organic compounds are saturated hydrocarbons, carboxylic acids, esters, alcohols, and some polymers, which are characterized by phase transition enthalpy in the range of 100–200 J/g and which are partly resistant to overheating or sub-cooling [19]. In addition, they are characterized by low fire resistance, low solid thermal conductivity, and relatively high prices. In turn, organic compounds are most often hydrated salts of the main groups of the periodic table, as well as some carbides, sulfides, and silicas. They are characterized by enthalpy of the phase transformation in the range of 150–350 J/g and are not resistant to overheating or sub-cooling. In addition, they are characterized by high fire resistance, good solid thermal conductivity, and relatively low prices. An undoubted disadvantage of this PCM group is the frequent phenomenon of irreversible separation of hydrate crystallization water from the solid part of the salt, which according to [20,21,22] is due to the sedimentation of the heavier particles to the bottom of the containers in which they are located. The last of the groups of phase change materials cited in the scientific literature are eutectic mixtures [17,18,23,24,25,26]. These are materials that under conditions of constant pressure, concentration of components, and temperature may occur in two or more physical states. They are usually mixtures of the above two PCM groups that are created to obtain materials possessing the advantages of organic and inorganic PCMs, while eliminating their disadvantages.

Considering the advantages and disadvantages of phase change materials, their applications in construction, and their planned application in research, the phase change material independently developed and produced by the authors described in [27] was used, which is the subject of a patent application [28]. This PCM, which is a eutectic mixture of two carboxylic acid esters, was chosen due to its melting and solidification enthalpy values and the invariability of its thermophysical properties, even after 2000 phase transformation cycles [19].

The use of phase change materials in construction has been the subject of numerous scientific studies since the 1970s [29,30,31,32,33], but many technical and scientific problems related to their economically justified use in construction and modeling of their thermal functions have not been resolved. These include:

- Incorrect selection of PCMs used in the application location in question;
- Unsealing forms moulds with PCMs applied;
- Change of physicochemical properties of PCMs after many phase transformation cycles;
- Insufficient ability of solid-state PCMs to conduct heat.

In the scientific literature [34,35], phase change materials are used directly in transparent building partitions to improve their energy function; as fillings for the inter-pane space [35,36,37,38]; as light permeable coatings [39]; as fillings for window shading systems, such as shutters or blinds [40,41,42,43,44]; or in heat accumulators installed within the double façade [45]. In addition, these types of materials have been tested by applying them inside glass blocks [46]. Phase change materials in the above places are used in the form of microcapsules and microgranules [47,48,49,50], in the form of composites with a stable shape SSPCM [51,52,53,54], in the form of capsules or packages coated with a PCM retaining the coating in the assumed place [19,51,55], or in direct form [40,41,56].

When conducting a search of the scientific literature on the modeling of the thermal function and heat storage using phase change materials, it can be seen that most studies refer to Stefan’s solution for the problem described in [6]. By knowing the PCM temperature field we can calculate the amount of heat stored in it, neglecting changes in the internal energy of the system caused by changes in the temperatures of the liquid and solid PCM. The above approach has become the starting point for numerous scientific papers in this field [6,7,57,58], but nevertheless it is dedicated to substances characterized by phase changes at the “point”. Unfortunately, according to [11], only some of the non-coherent hydrates of inorganic salts with a very ordered crystal structure are characterized by the above properties. Therefore, modeling of heat storage in phase change materials is done by determining the temperature field using glued functions, in which the changing heat capacity values when the material is in a solid state, liquid, or during phase transformation are written separately. The weakest point of this approach is the appropriate mapping of the temperature field in the phase transition. As in the previous case, the limit is the temperature range at which the PCM phase transformation takes place. The above approach according to [59,60] is used for organic and inorganic PCMs, which are characterized by their high purity, resulting in a narrow temperature range for their phase transitions. The problem is the modeling of heat storage and distribution in PCMs that are characterized by a wide range of phase transition temperatures (e.g., 10–15 °C). At the same time, they are the cheapest and easily available compounds and mixtures enabling the expected thermal benefits under selected conditions.

The references cited in the literature present the results of research and analysis of selected application cases and specific phase change materials, showing their potential advantages and disadvantages. However, no general rules and relationships have been presented that would allow simulation of the thermal behaviour of a greater number of PCMs in a single solution, or a specific solution containing a specific PCM that would function in different climatic conditions around the world. For this reason, the authors’ method of conducting investigative experiments together with the development of a mathematical model with wide application possibilities is a complete solution to the studied problem, while allowing simulation of the thermal effects of the tested solution in other climatic conditions.

## 2. Materials and Methods

### 2.1. Materials

- Phase change material (self-produced eutectic mixture of two esters: propyl palmitate and butyl stearate). The method of production as well as physical and chemical properties are presented in [27,28];
- 2 mm thick aluminum sheet, covered with matt black paint.

### 2.2. Apparatus

- Almemo 2890-9 recorder, company Ahlborn, Ilmenau, Germany;
- Pt1000 temperature sensor, company Salus, Kobielice, Poland;
- Heat flux density sensor: FQA020C, company Ahlborn, Ilmenau, Germany;
- LT 019008 thermocouple, company Ahlborn, Ilmenau, Germany;
- Almemo FLA 613 GS pyranometer, company Ahlborn, Ilmenau, Germany.

### 2.3. Resarch Method

In the discussed problem, energy efficiency is understood as the ratio of the heat balance of the transparent partition with PCM to the heat balance of the reference transparent partition. In both cases, the heat balance was determined as the least-squares integral recorded on the internal glazing of the examined windows for heat flux density values as a function of time, according to Equation (1).

(1) $$Q_{window}=\int_{t=1}^{t=n}q_{t}dt$$

where *t* is time.

Another physical quantity used in the work to demonstrate a change in the energy efficiency of the tested windows was the number of degree-hours. The number of degree-hours is understood to be the sum of the differences in the glazing temperature and the planned air temperature inside the chamber that is maintained for a given time. As such, the smaller the number of degree-hours, the better the adjustment of the heat gains generated by the window to the heat demand profile of the adjacent room.

(2) $$S_{\mathrm{th}}=\overset{t=n}{\underset{t=1}{\sum }}\left|\left(T_{1}-T_{0}\right)\right|$$

Assessments of the obtained results for heat balance and the number of degree-hours were carried out in terms of short and long periods of time.

### 2.4. Experimental Tests

**Figure 1:**
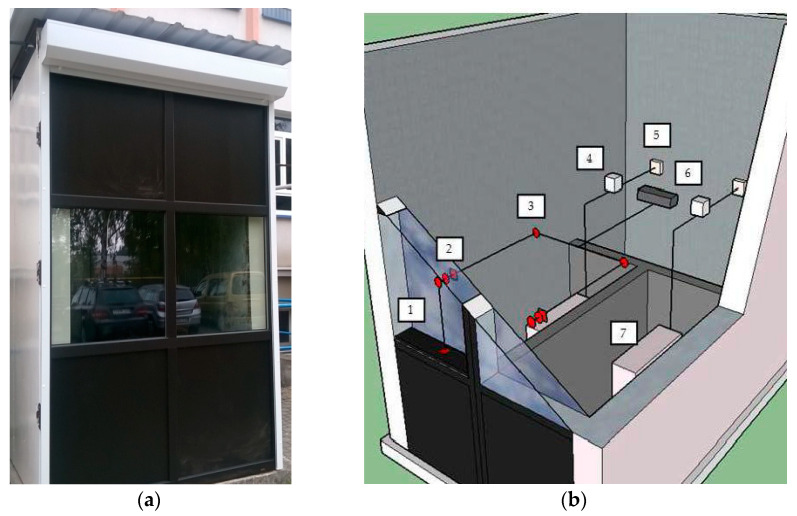
(**a**) Photograph of the isothermal field testing chamber. (**b**) Diagram of the field testing chamber: (1) heat accumulator; (2) heat meter with thermocouple; (3) temperature sensor; (4) thermoregulator; (5) energy consumption meter; (6) recorder; (7) oil heater.

The experimental tests were conducted in an isothermal field testing chamber, functioning in real conditions of the external climate. The research was carried out during the heating season (from October to April) in Rzeszów in Poland. The field testing chamber had a steel skeleton structure thermally insulated with a 20 cm layer of mineral wool. The chambers external dimensions were 280 cm long, 190 cm wide, and 270 cm high. From the south side of the chamber, two identical windows with dimensions of 90 cm × 60 cm were installed (Figure 1). The interior of the chamber was divided into two identical volumes, in which identical windows, oil heaters, thermoregulators, and electronic meters of electricity consumption were installed. The windows in both parts of the chamber consisted of two glazing units, creating a 15 cm space between them. Within the space between the panes of one of the windows, a heat accumulator was placed in the form of an aluminium box measuring 650 mm × 100 mm x 50 mm, inside which phase change material was applied. The material used is a eutectic mixture of propyl palmitate and butyl stearate, developed and produced by the author of [27]. During the tests, the intensity of solar radiation was recorded along with the temperatures of internal and external air and the internal panes in both windows. In addition, the density of the heat flux penetrating the glazing and the amount of electricity needed to supply oil heaters were recorded. Data were recorded simultaneously in both parts of the chamber at 10 min time intervals. This research is a continuation and extension of the research carried out by the authors and described in [19,27,28,40,41] on the possibilities of the effective use of thermal gains from solar radiation to reduce the cost of heating buildings.

As part of the described tests, the thermal functions of 6 combinations of glazing units in a window with phase changes and reference materials were tested. The parameters of all glazing units used during the tests are given in Table 1. The testing of each combination lasted between 1 and 1.5 months.

The conducted experiments allowed the creation of a database that was used to validate and verify the mathematical model. This will be a useful tool in determining the potential thermal gains associated with the application of a discussed solution with an innovative phase change material in any outdoor climate conditions.

### 2.5. Mathematical Model

A model was made using equations of non-stationary heat flow and an explicit finite difference method. The model consisted of two connected parts with an identical time interval length of 10 min. One part of the model describes one-dimensional heat flow within a window combined with a heat accumulator, while the other part describes two-dimensional heat flow and storage in a phase change heat accumulator. The general heat flow formulas and time interval lengths are presented below:

(3) $$T_{2}^{t+1}=\frac{\Delta t}{C_{w2}\cdot \rho_{2}}\cdot \left(\frac{T_{1}^{t}-T_{2}^{t}}{R_{1-2}}+\frac{T_{3}^{t}-T_{2}^{t}}{R_{2-3}}\right)+T_{2}^{t}$$

where is the Δ*t* time interval, *C_w2_* is the specific heat of the material at point 2, *ϱ_2_* is the density at point 2, *R_1-2_* is the heat resistance between points 1 and 2, and *T^t^_1_* is the temperature at point 1 during t.

In turn, the determination of the two-dimensional map of the temperature fields inside the accumulator with the PCM in the time step t + 1 was determined by the formula:

(4) $$T_{\text{PCM}.2,2}^{t+1}=\frac{\Delta t}{eff_{C.PCM}\cdot \rho_{\text{PCM}}}\cdot \left(\begin{array}{c} \frac{T_{\text{PCM}.2,1}^{t}-T_{\text{PCM}.2,2}^{t}}{R_{\text{PCM}.2,1-2,2}}+\frac{T_{\text{PCM}.2,3}^{t}-T_{\text{PCM}.2,2}^{t}}{R_{\text{PCM}.2,2-2,3}}+\\ \frac{T_{\text{PCM}.1,2}^{t}-T_{\text{PCM}.2,2}^{t}}{R_{\text{PCM}.1,2-2,2}}+\frac{T_{\text{PCM}.3,2}^{t}-T_{\text{PCM}.2,2}^{t}}{R_{\text{PCM}.3,2-2,2}} \end{array}\right)+T_{\text{PCM}.2,2}^{t}$$

where Δ*t* is the time interval, *eff_C.PCM_* is the specific heat of the PCM, *ϱ_PCM_* is the density PCM, and *R*_1,2-2,2_ are the heat-resistant PCM values between points 1,2 and 2,2.

(5) $${{\text{T}}^{t}}_{2,2}\;\text{temperature PCM at point 2,2 during t}\;\Delta t\leq min\left[\frac{\Delta x_{i}^{2}}{2\;\cdot \;\frac{\lambda_{i}}{\rho_{i}\;\cdot \;C_{w,i}}}\right]$$

where $\;\Delta x_{i}^{2}$ is the square of the element thickness i $,\;C_{w,i}$ is the specific heat of the material at i , ρ_i_ is the density at point I, and *λ_i_* is the heat transfer coefficient i.

**Figure 2:**
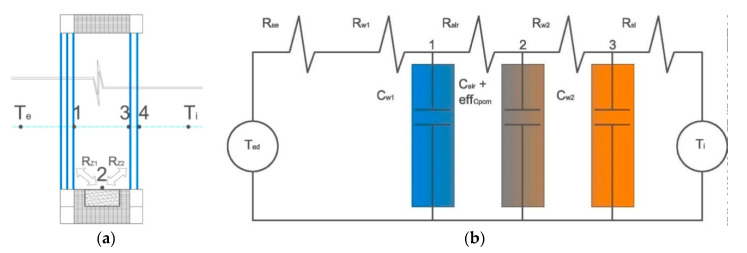
(**a**) Graphical diagram of the mathematical model of a composite window with a phase change heat accumulator. (**b**) Analogous electric diagram of the mathematical model of a composite window with a phase change heat accumulator.

The graphical and analogous electric diagram of the mathematical model is presented below in Figure 2.

The transmittance of heat and solar radiation through a composite window is a complex issue that has been dealt with in numerous scientific papers [6,7,8,9,10,49,50,51]. When creating this part of the model, complex heat exchange by conduction, convection, and radiation between a window’s individual glazing was taken into account. The essence of the solution to this problem is to determine two complex heat transfer resistances between the external glazing and the PCM accumulator (R_Z1_), and between the PCM accumulator and the internal glazing (R_Z2_). These complex heat resistances are temperature functions that take into account heat transfer by convection q_k_ and radiation q_r_. The above thermophysical phenomena were considered in accordance with the relationships described in [61].

(6) $$q_{z}=q_{k}+q_{r}$$

(7) $$q_{k}=\frac{\left(t_{1}-t_{2}\right)\lambda }{d}$$

(8) $$q_{r}=h_{r}\left(t_{1}-t_{2}\right)$$

where *q_z_* is the heat flux density transmitted by radiation and convection, *q_k_* is the heat flux density transmitted by convection, and *q_r_* is the flux density transmitted by radiation.

Performing formula substitutions and transformations for Equations (6), (7), and (8), Equation (9) was obtained in accordance with [61]:

(9) $$R_{z}=\frac{t_{1}-t_{2}}{q_{z}}=\frac{1}{\frac{h_{r}}{d}+h_{r}}$$

Using the Jacob formula described by Pogorzelski in [61], the values of the substitute heat transfer coefficient for fluids *λr* were determined:

(10) $$\frac{\lambda_{r}}{\lambda }=F(Gr,\mathrm{Pr})$$

Grashof’s probability number is:

(11) $$\begin{array}{c} \frac{\lambda_{r}}{\lambda }=1|for\;Gr<10^{3}\\ \frac{\lambda_{r}}{\lambda }=18\cdot 10^{-2}{\left(Gr\right)}^{\frac{1}{4}}{\left(\frac{\delta }{H}\right)}^{\frac{1}{9}}|for\;\;2\cdot 10^{4}<Gr<2\cdot 10^{5}\\ \frac{\lambda_{r}}{\lambda }=65\cdot 10^{-3}{\left(Gr\right)}^{\frac{1}{3}}{\left(\frac{\delta }{H}\right)}^{\frac{1}{9}}|for\;\;2\cdot 10^{5}<Gr<11\cdot 10^{6} \end{array}$$

where *δ*, *H* are the width and height of the air gap.

Grashof and Prandtel probability numbers, according to [61], are expressed by the formulas presented below:

(12) $$Gr=\;\frac{\beta \cdot g\;\cdot \;l^{3}\cdot \Delta T}{v^{2}}$$

(13) $$Pr=\frac{v}{a}$$

where *β* is the air thermal expansion coefficient, *g* is the gravitational acceleration, *v* is the kinetic coefficient of the air viscosity, *a* is the temperature equalization coefficient, and ∆*T* is the temperature and air difference at a distance.

Radiation heat exchange in the unventilated space between the panes was taken into account according to Equation (14):

(14) $$h_{r}=\frac{q_{1-2}}{T_{1}-T_{2}}=\;\varepsilon_{1-2}\;\cdot \;C_{0}\;\cdot \;\varphi_{1-2}\left[\frac{{\left(\frac{T_{1}}{100}\right)}^{4}-{\left(\frac{T_{2}}{100}\right)}^{4}}{T_{1}-T_{2}}\right]$$

where *C*_0_ is the black body radiation factor, *ε*_1-2_ is the replacement emissivity, and *φ*_1-2_ is the angle radiation factor (so-called configuration factor).

Due to the fact that heat exchange by radiation occurs at a right angle between the surface of the heat accumulator absorber *F*_1_ and glazing surface *F*_2_, the equivalent emissivity $\varepsilon_{1-2}$ and the angular radiation factor *φ*_1-2_ were determined in accordance with Equations (15) and (16):

(15) $$\varepsilon_{1-2}=\frac{1}{\frac{1}{\varepsilon_{1}}+\frac{F_{1}}{F_{2}}\left(\frac{1}{\varepsilon_{2}}\;-\;1\right)}$$

(16) $$\varphi_{1-2}=\frac{1}{F_{1}}\int_{F_{1}}^{N}\int_{F_{2}}^{N}\frac{\cos\beta_{1}\cos\beta_{2}}{\pi \;\;R^{2}}dF_{1}dF_{2}$$

On the other hand, the heat flow and distribution within the phase change heat accumulator was determined based on Equation (3) in two-dimensional terms and the glued function of enthalpy increase inside the PCM as a function of temperature, in accordance with Equation (17). The above approach was used in numerous scientific works [6,56,57].

(17) $$eff_{C.PCM}=\Delta H_{PCM}=\{\begin{array}{cc} m_{s}\cdot C_{W.S}\cdot \left(T_{L}-T_{0}\right) & for\;\;\;\;\;\;\;\;T>T_{L}\\ m_{L}\cdot \Delta H_{L} & for\;\;\;\;\;\;\;\;\;T=T_{L}\\ m_{l}\cdot C_{W.l}\cdot \left(T_{l\;}-T_{L}\right) & for\;\;\;\;\;\;\;\;T<T_{L} \end{array}$$

The functions of the change of PCM mass enthalpy during *H_PCM_* phase transformation over time were developed using the calorimetric thermogram obtained and described by the author [27]. The above approach is analogous to solving Stefan’s problem [6,59], except that it concerns compounds with blurred melting and solidification. For the needs of this model, a discrete accumulator grid from PCM was selected with a width of *dx* = *dy* = 5 mm. Therefore, the cross-section of the accumulator in question consisted of 200 elements (grid dimensions: 50 mm × 100 mm). In addition, the extreme nodes of the discussed cross-section were modelled as the aluminium side of the accumulator, taking into account the resistance and heat capacity of the aluminium alloy used in the field tests.

## 3. Results

### 3.1. Experimental Results

**Figure 3:**
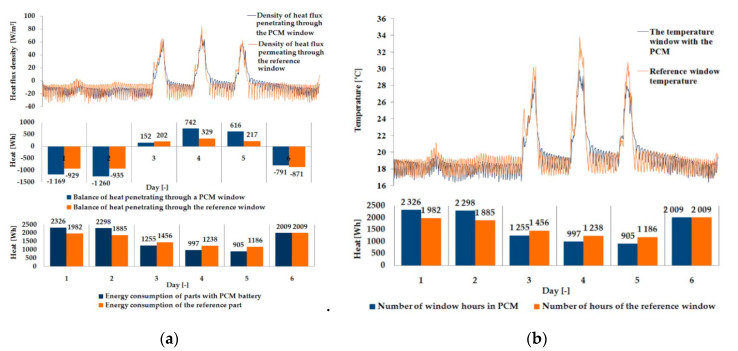
Thermal functioning of the window with an external triple glazing unit and an internal single glazing unit. (**a**) Graph of the heat flux density values recorded on the inner surface of the glazing unit with the PCM and the reference window. (**b**) Graph of temperature values recorded on the inner surface of the PCM glazing unit and the reference window.

**Figure 4:**
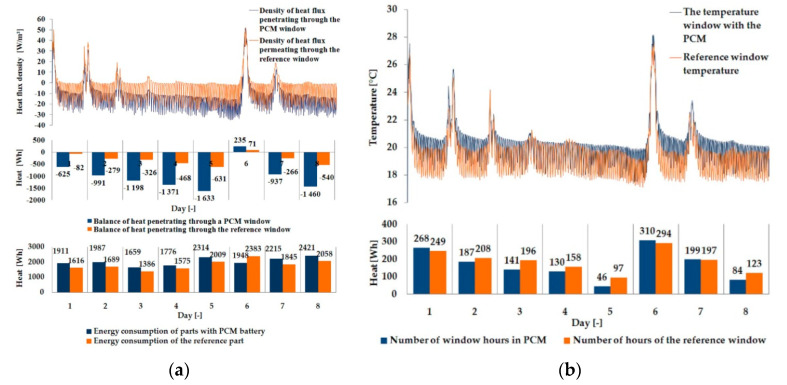
Thermal functioning of the window with an external triple glazing unit and an internal double glazing unit. (**a**) Graph of heat flux density values recorded on the inner surface of the glazing unit with the PCM and the reference window. (**b**) Graph of temperature values recorded on the inner surface of the PCM glazing unit part and the reference window.

**Figure 5:**
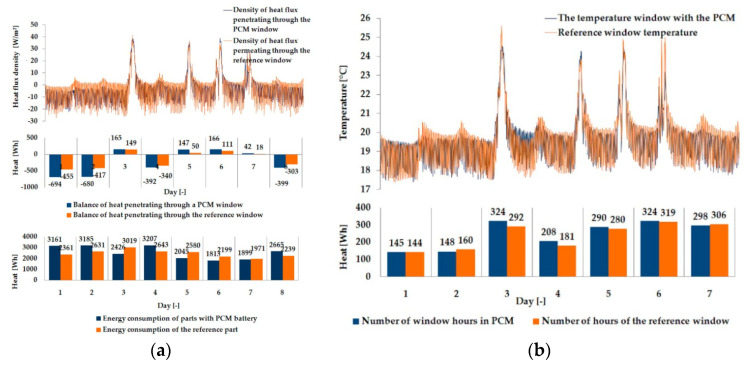
Thermal functioning of the window with an external triple glazing unit and internal triple glazing unit. (**a**) Graph of heat flux density values recorded on the inner surface of the glazing unit with the PCM and the reference window. (**b**) Graph of temperature values recorded on the inner surface of the PCM glazing unit and the reference window.

**Figure 6:**
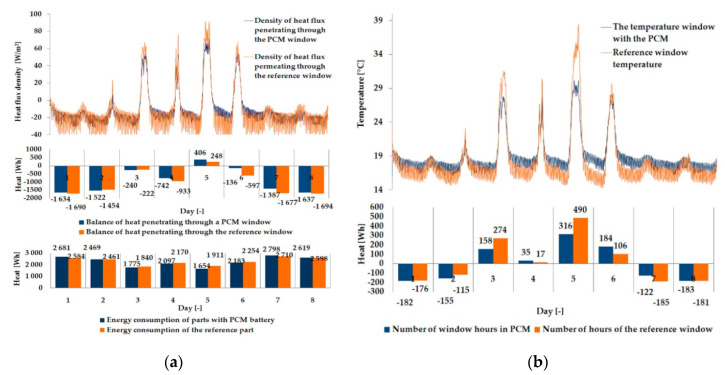
Thermal functioning of the window with an external double glazing unit and an internal single glazing unit. (**a**) Graph of heat flux density values recorded on the inner surface of the glazing unit with the PCM and the reference window. (**b**) Graph of temperature values recorded on the inner surface of the PCM glazing unit and the reference window.

**Figure 7:**
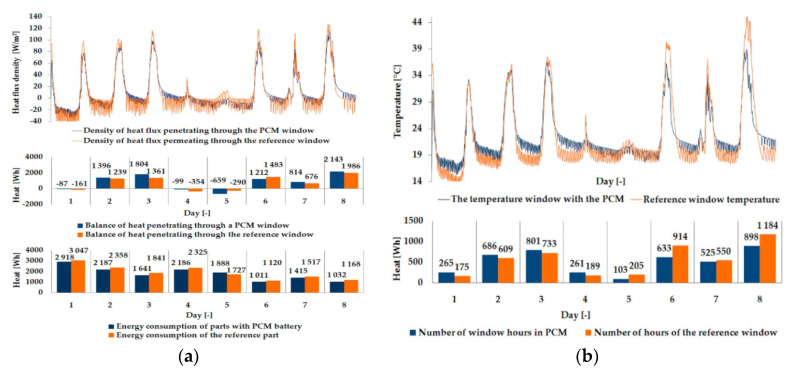
Thermal functioning of the window with an external double glazing unit and an Internal double glazing unit. (**a**) Graph of heat flux density values recorded on the inner surface of the glazing unit with the PCM and the reference window. (**b**) Graph of temperature values recorded on the inner surface of the PCM glazing unit and the reference window.

**Figure 8:**
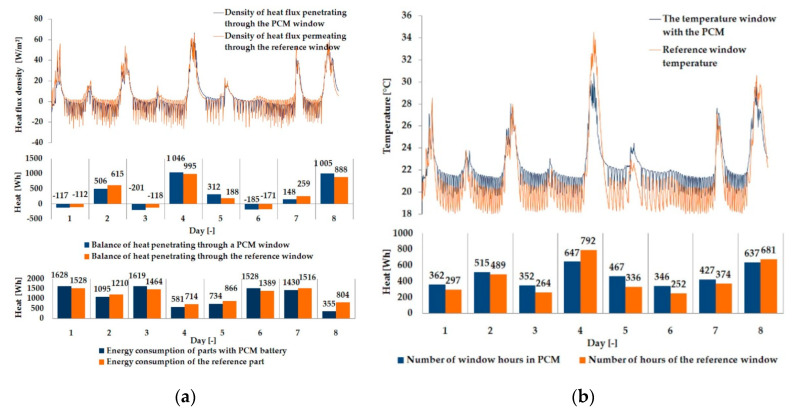
Thermal functioning of the window with an external double glazing unit and an internal triple glazing unit. (**a**) Graph of heat flux density values recorded on the inner surface of the glazing unit with the PCM and the reference window. (**b**) Graph of temperature values recorded on the inner surface of the PCM glazing unit and the reference window.

The empirical results of heat flux density and temperature obtained during the tests, recorded on the inner surface of the internal glazing, are summarized below in the form of line and bar charts (Figure 3, Figure 4, Figure 5, Figure 6, Figure 7 and Figure 8). This allows observation of both the momentary waveforms of the PCM and the reference values recorded in the modified windows, as well as their daily totals.

In Figure 3, Figure 4 and Figure 5, one can observe a diminishing influence of PCM on the instantaneous temperature and flux density distributions of heat passing through the window when comparing the modified window to the reference window. With an increase in heat resistance and a decrease in the transmissivity of the internal glazing, the phase shift of the curve describing the PCM window becomes less visible in relation to the curve describing the reference window. There is also a visible trend of reductions of peak value differences during both sunny and cloudy days of temperature values and heat flux density for the window with PCM compared to the reference window value.

In combination with the external double-glazed units shown in Figure 6, Figure 7 and Figure 8, as in the previous case with the external triple-glazed unit, there is a noticeable reduction in the differences in peak temperature values and the heat flux density of the PCM window and the reference window, although not to such an extent. Additionally, with an increase in the number of internal window panes, there was no significant reduction in the phase shift of the graphs relating to the PCM window compared to the reference window.

The results presented above in Figure 3, Figure 4, Figure 5, Figure 6, Figure 7 and Figure 8 for each of the six combinations of glazing units examined during sunny days showed that glazing with PCM was characterized by higher daily heat balances, smaller daily degree-hours, and lower daily heat demand from external sources than reference glazing. On the other hand, during cloudy days, PCM glazing was characterized by lower daily heat balances, higher daily degree-hours, and a greater daily demand for heat from external sources than reference glazing. The above information proves that during cloudy days in the heating season, the PCM heat accumulator may adversely affect the function of the modified PCM window.

### 3.2. Results of the Mathematical Simulation

**Figure 9:**
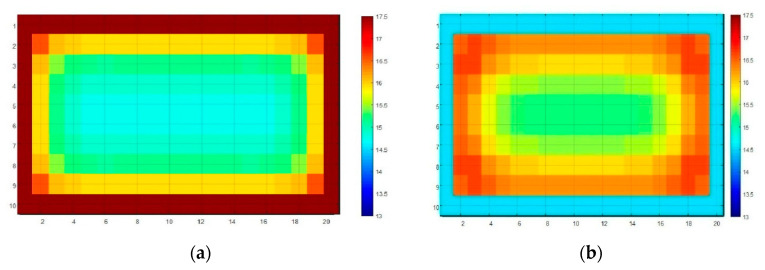
Results of the mathematical simulation. (**a**) Accumulator temperature field with PCM during charging. (**b**) PCM accumulator temperature field during discharging.

The following are the cross-sectional temperature fields of the heat accumulator obtained thanks to the author’s phase change material. Figure 9 shows the PCM temperature distributions during accumulator charging and discharging.

**Figure 10:**
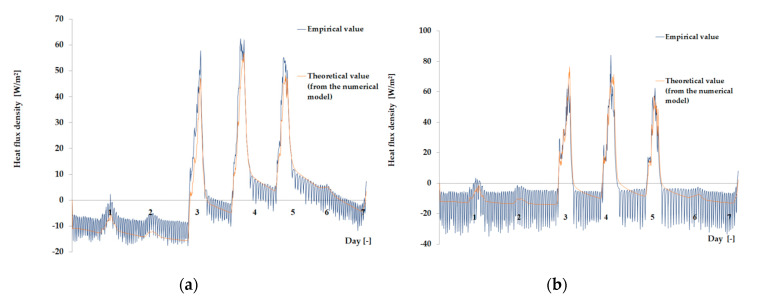
Results for the external triple glazing unit and internal single glazing unit. (**a**) Comparison of heat flux density values recorded and obtained from the model in the PCM part. (**b**) Comparison of the density values of heat fluxes registered and obtained from the model in the reference part.

**Figure 11:**
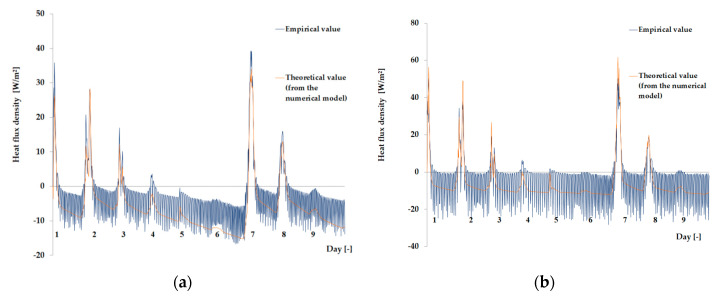
Results for the external triple glazing unit and internal double glazing unit. (**a**) Comparison of heat flux density values recorded and obtained from the model in the PCM part. (**b**) Comparison of the density values of heat fluxes registered and obtained from the model in the reference part.

**Figure 12:**
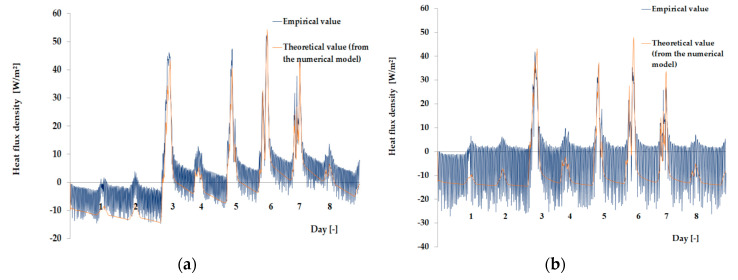
Results for the external triple glazing unit and internal triple glazing unit. (**a**) Comparison of heat flux density values recorded and obtained from the model in the PCM part. (**b**) Comparison of the density values of heat fluxes registered and obtained from the model in the reference part.

**Figure 13:**
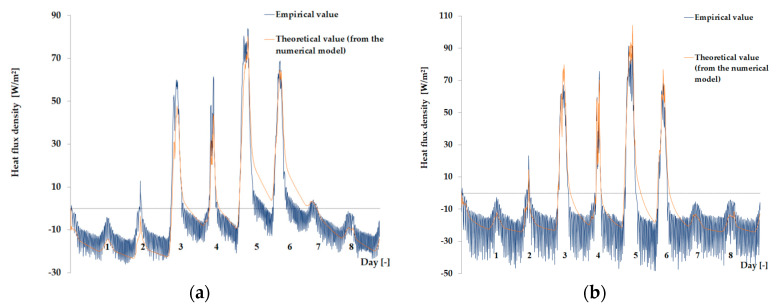
Results for the external double glazing unit and internal single glazing unit. (**a**) Comparison of heat flux density values recorded and obtained from the model in the PCM part. (**b**) Comparison of the density values of heat fluxes registered and obtained from the model in the reference part.

**Figure 14:**
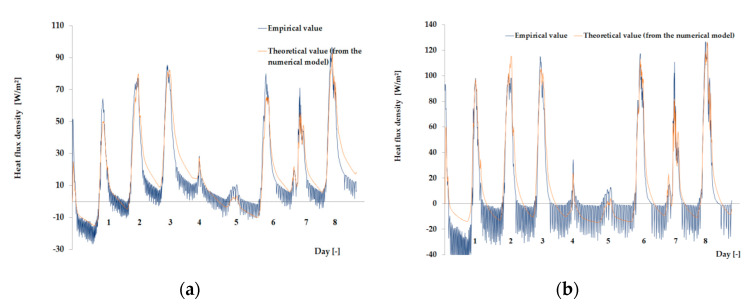
Results for the external double glazing unit and internal double glazing unit. (**a**) Comparison of heat flux density values recorded and obtained from the model in the PCM part. (**b**) Comparison of the density values of heat fluxes registered and obtained from the model in the reference part.

**Figure 15:**
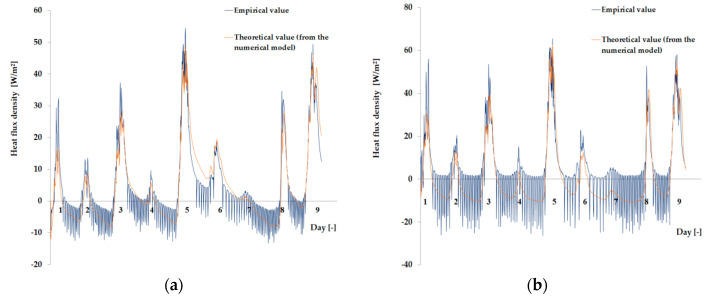
Results for the external double glazing unit and internal triple glazing unit. (**a**) Comparison of heat flux density values recorded and obtained from the model in the PCM part. (**b**) Comparison of the density values of heat fluxes registered and obtained from the model in the reference part.

As the amount of heat stored in the accumulator was known thanks to the developed mathematical model, the theoretical results of the thermal functioning of the modified PCM window were obtained. Figure 10, Figure 11, Figure 12, Figure 13, Figure 14 and Figure 15 below are graphs that allow observation of the adjustment of empirical and theoretical values for the temperature and heat flux density. The results apply to all 6 combinations of glazing units considered.

Figure 10, Figure 11, Figure 12, Figure 13, Figure 14 and Figure 15 show a good fit of empirical and theoretical data. The most visible differences in matching both data sets are in the combinations in which the external glazing consists of a double-pane window (Figure 13, Figure 14 and Figure 15). In these cases, it can be seen that the representation of the empirical results by the model in the evening period after sunny days is not very accurate.

### 3.3. Statistical Analysis

The assessment of the adequacy of the model performed in relation to empirical data was carried out by performing a statistical test used to compare the variance of two populations of results, the Snedecor–Fischer test. As part of this test, the result of the null hypothesis was checked, with an assumed error rate of 5%. The obtained results of all considered glazing combinations are presented in Table 2.

The results of the statistical tests showed that in the case of two combinations of glazing, the error of the verified model did not fall within the assumed 5% range. In addition, the fact that the Snedecor–Fischer test statistics were susceptible to peak measurement values required additional verification of the mathematical model. This was carried out by analysing the dispersion of empirical quantities relative to those simulated using the quasi-Newton method. Considering the results of statistical tests, the quasi-Newton fit analysis was performed on four cases satisfying the null hypothesis of the Snedecor–Fischer test. The analysis of the fit was carried out by determining the minimum error function as the sum of the squares of the differences of the values measured experimentally *T_d_(t*), obtained from the mathematical calculations *T_s_(t)*.

(18) $$\min y=\overset{n}{\underset{t=1}{\sum }}{\left[T_{d}\left(t\right)-\;T_{s}\left(t\right)\right]}^{2}$$

The determination of the minimum error function and the values of the coefficients of determination are described by the calculated values. The determined values of the determination coefficient were made in accordance with Equation (19), using MS Excel with the add-on solver.

(19) $$R^{2}=1-\frac{\sum_{t=1}^{n}{\left({\hat{y}}_{t}-\bar{y}\right)}^{2}}{\sum_{t=1}^{n}{\left(y_{t}-\bar{y}\right)}^{2}}$$

where *y_t_* is the actual value of *y* at *t* $,\;{\hat{y}}_{t}$ is the theoretical value of the explanatory variable, and $\;\bar{y}$ is the arithmetic mean of the values of the explanatory variable.

**Figure 16:**
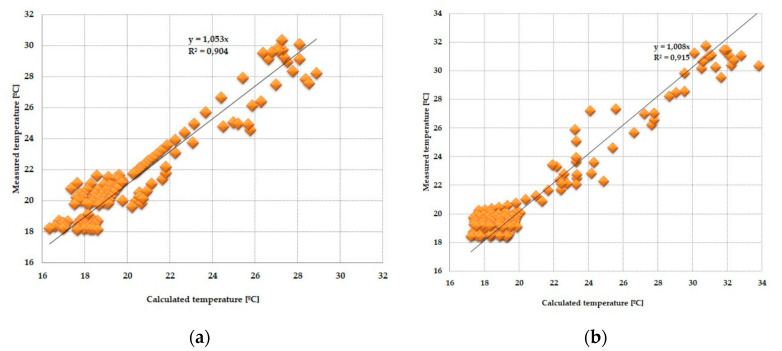
(**a**) Adjustment of the temperature value of the internal pane, measured relative to those calculated in the PCM accumulator part, with an external triple-pane window and an internal single-pane window. (**b**) Adjustment of the temperature values of the internal pane, measured relative to those calculated in the reference part, with an external triple-pane window and an internal single-pane window.

**Figure 17:**
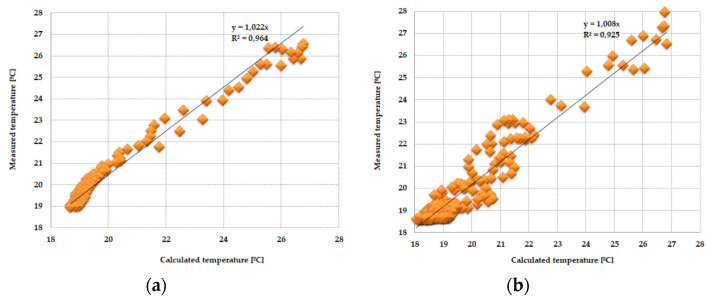
(**a**) Adjustment of the internal temperature value of the pane, measured relative to those calculated in the PCM accumulator part, with an external triple-pane window and an internal double-pane window. (**b**) Adjustment of the temperature value of the internal pane, measured relative to those calculated in the reference part, with an external triple-pane window and an internal double-pane window.

**Figure 18:**
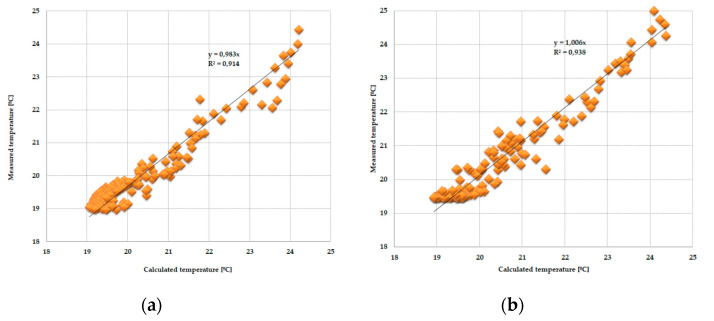
(**a**) Adjustment of the internal temperature value of the pane, measured relative to those calculated in the PCM accumulator part, with an external triple-pane window and an internal triple-pane window. (**b**) Adjustment of the temperature value of the internal pane, measured relative to those calculated in the reference part, with an external triple-pane window and an internal triple-pane window.

**Figure 19:**
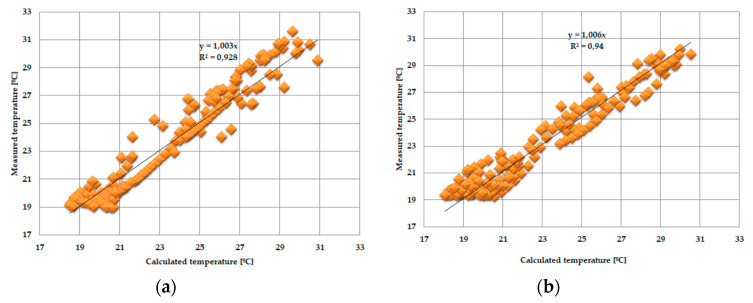
(**a**) Adjustment of the internal temperature value of the pane, measured relative to those calculated in the PCM accumulator part, with an external double-pane window and an internal triple-pane window. (**b**) Adjustment of the temperature value of the internal pane, measured relative to those calculated in the reference part, with an external double-pane window and an internal triple-pane window.

One of the possible ways to assess the fit of measured values relative to calculated values may be to determine the relationship between them and the linear function *y = ax*, shown in Figure 16, Figure 17, Figure 18 and Figure 19.

The obtained results from the analysis of the matching of the empirical and theoretical data indicate their good fit. This is evidenced by the values of the “*a*” direction coefficients and *R^2^* determination close to unity. There was also no significance in the expression of free linear regression equations in the cases considered.

### 3.4. Simulations of the Operation of a Composite Window with a Phase Change Heat Accumulator for Data of a Typical Meteorological Year in Rzeszów

**Figure 20:**
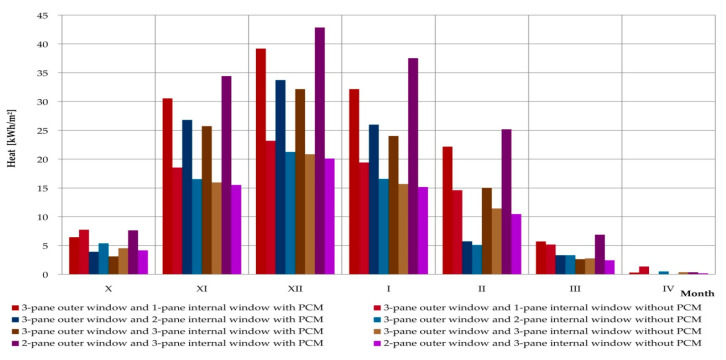
Graph of heat loss of a composite window with a phase change cushion according to data for a typical meteorological year in Rzeszów.

**Figure 21:**
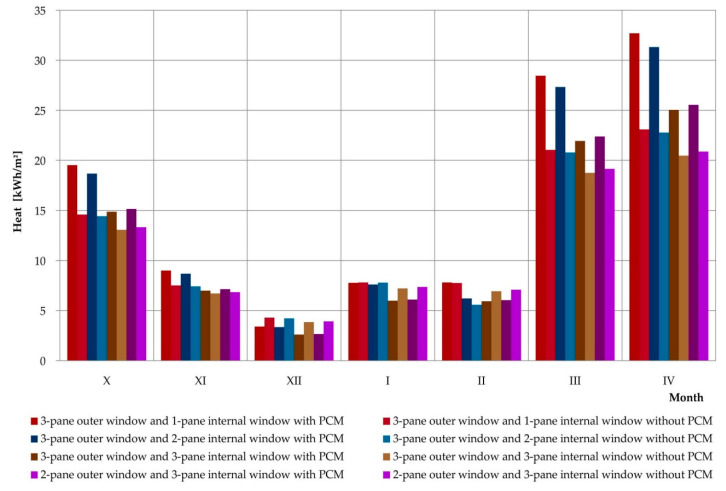
Graph of the heat gain of a composite window with a phase change cushion according to data for a typical meteorological year in Rzeszów.

**Figure 22:**
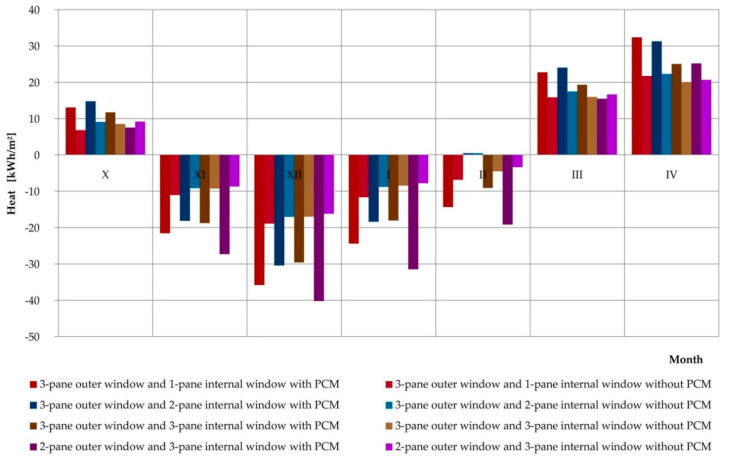
Graph of heat balance results for a composite window with a phase change cushion according to typical data for a meteorological year in Rzeszów.

An additional utilitarian goal of the experimental research and mathematical analyses was to perform simulations of profits, losses, and heat balance for the considered composite windows with phase change heat accumulators and the reference window. The simulation was carried out thanks to a positively verified mathematical model, using hourly data for a typical meteorological year for the nearest meteorological station. The simulations covered the entire heating season, during which 4 positively verified combinations of glazing units were considered. The obtained results are presented in Table 3 and graphically in Figure 20, Figure 21 and Figure 22, separately comparing the gains, losses, and heat balance generated by the considered reference windows and the windows modified with PCM accumulators.

The simulation results prove that except for one case (window with a triple external glazing unit and single internal glazing unit in October) each month, each combination of glazing with a variable phase heat accumulator generated greater heat loss than their reference windows. However, in most cases the values of losses generated exceeded the values of profits generated. This adversely affected the values of the average monthly heat balances of PCM-modified windows relative to reference ones. Analyzing the data contained in Figure 22, we can see the more favourable monthly values for heat balance with PCM-modified windows compared to reference windows in spring and autumn, for example in October, March, and April. This clearly proves the thermal benefits resulting from the application of the considered solution on a monthly basis, and also when considering locations with a warmer climate on a seasonal basis.

## 4. Discussion

Comprehensive, extensive all-season field tests in a large-scale isothermal chamber with 6 different combinations of building window glazing units allowed clear evidence to be obtained of the improved thermal performance of composite windows with a phase change heat accumulator. The presented results of the experimental studies have proven the impacts of using a phase change heat accumulator on the thermal efficiency of a composite window. The above function was proved via both the temperature and density of the heat flux recorded on the inner surface of the glazing, as well as the recorded energy consumption for heating both parts of the field testing chamber.

There was a decrease in PCM glazing peak temperatures and a time shift in the charts for the recorded temperature and heat flux density during sunny days for each of the combinations tested. Nevertheless, the PCM heat accumulator was not subject to the thermal functioning of the composite window during cloudy days and contributed to the increase of the window heat loss. This allowed exclusion of some climate zones as being economically unjustified in terms of applying the tested solution.

The presented mathematical model, implemented on the basis of non-stationary heat flow equations of the finite difference method and taking into account complex heat exchange, underwent verification of its statistical adequacy through the F (Snedecor) test, using experimental data for 4 out of 6 tested glazing combinations. The two cases that did not meet the conditions of the null hypothesis and the alternative test were the combinations of single and double glazing units with high thermal diffusivity. For these and similar cases, better-matched models should be developed using non-simplified thermodynamics and fluid mechanics equations.

The positively verified combinations of glazing units can be successfully used to conduct analyses of the impacts of the tested solutions on a window’s thermal balance in various climatic conditions due to the very good matching of empirical and theoretical results, as evidenced by the close unity of the values obtained for direction and determination coefficients and the quasi-Newton analysis of fit. The results of the analysis conducted on the basis of data from a typical meteorological year for the climate of Rzeszów (Poland) prove that in the considered location the tested solution causes a decrease in the heat balance of the entire heating season.

An improvement in the adjustment of heat gains from solar radiation to the heat demand profile of the internal glazing of the PCM window was recorded in comparison with the reference window for the combination of an external triple glazing unit and internal single glazing unit.

In terms of the entire heating season, the smallest reduction in the heat balance of the PCM window relative to the reference window was noted for the combination of an external triple glazing unit and internal double glazing unit. In turn, the largest decrease was noted with the combination of an external double glazing unit and internal triple glazing unit.

The above facts are a result of the nature of the climate in question, where there are approximately 100 sunny days a year on average. Therefore, in order to improve the heat balance of the window, this solution with the proposed phase change material should be used in a slightly warmer climate, e.g., moderate warm or Mediterranean. The above empirical and theoretical results, as well as the analyses carried out and the cognitive values, have enabled the creation of a tool that can be used in the design of new buildings and in thermomodernization of existing ones.

## 5. Conclusions

The implementation and dissemination of the tested solution has a chance of success due to rising energy prices and the increase in ecological awareness of societies. While the prices of organic phase change materials have dropped significantly compared to recent years, technological problems related to the effective application of PCM in the accumulator need to be solved. Another aspect that requires additional research is the increase in the intensity of charging and heat reception from the accumulator over time. An advantage of the proposed solution is the easy and relatively inexpensive modernization of the windows of existing buildings by installing additional glazing on the inside of the window and an accumulator with a PCM in the space created between the panes. The next steps in the implementation of this solution should be the development of an inexpensive modular system for installing a heat accumulator, with the lower part being thermally insulated. In addition, software should be made available to companies producing windows so that at the design stage this solution, along with information about the thermal and financial benefits associated with its application, can be offered to potential buyers.

## 6. Patents

1. Lichołai L., Musiał M., Szyszka J. Mobile window insulation nr: EP.15461528.0 from 04.05.2015.

2. Musiał M. Phase-change material and method of producing phase-change material nr: P.425190 from 12.04.2018.

## Figures and Tables

**Table 1:** List of glazing parameters used during tests.

Parameter	Single Glazing	Double Glazing Unit	Triple Glazing Unit
Heat transfer coefficient (W/m^2^·K)	5	1.1	0.7
Transmittance (-)	0.82	0.75	0.5
Light permeability (-)	0.89	0.78	0.6
Construction of glazing unit (mm)	/4/	/4/16 Ar/4/	/4/16Ar/4/16 Ar/4/

**Table 2:** List of glazing parameters used during tests.

Sets of Glazing Units	Quotient of Variance for PCM Part	Critical Value	Quotient of Variance for the Reference Part	Critical Value
	**F_PCM (α,f1,f2)_**	**F_kr_**	**F_Ref (α,f1,f2)_**	**F_kr_**
3–1	1.0485	1.1100	1.0306	1.1100
3–2	1.1021	1.1100	1.0213	1.1100
3–3	1.1027	1.1100	1.0900	1.1100
2–1	1.2242	1.1100	1.0337	1.1100
2–2	1.2241	1.1100	1.1000	1.1100
2–3	1.0943	1.1100	1.0155	1.1100

**Table 3:** Heat balance of the tested combinations of windows, according to the data for a typical meteorological year in Rzeszów.

Exterior Window	Triple Glazing Unit						Double Glazing Unit	
Inner Window	Single Glazing		Double Glazing Unit		Triple Glazing Unit		Triple Glazing Unit	
	Window with PCM	Reference Window	Window with PCM	Reference Window	Window with PCM	Reference Window	Window with PCM	Reference Window
Month	(kWh)							
X	13.06	6.82	14.73	9.03	11.71	8.510	7.49	9.13
XI	−21.57	−11.04	−18.13	−9.14	−18.74	−9.254	−27.29	−8.70
XII	−35.81	−18.91	−30.39	−17.03	−29.56	−17.008	−40.21	−16.20
I	−24.39	−11.63	−18.38	−8.78	−18.05	−8.472	−31.44	−7.82
II	−14.37	−6.87	0.46	0.47	−9.11	−4.524	−19.15	−3.40
III	22.71	15.85	23.99	17.44	19.29	15.970	15.47	16.67
IV	32.36	21.72	31.26	22.26	25.02	20.073	25.17	20.67
Sum	−19.45	5.30	3.55	14.25	−−19.45	5.295	−69.97	10.36
